# Development, Application, and Performance of Artificial Intelligence in Cephalometric Landmark Identification and Diagnosis: A Systematic Review

**DOI:** 10.3390/healthcare10122454

**Published:** 2022-12-05

**Authors:** Nuha Junaid, Niha Khan, Naseer Ahmed, Maria Shakoor Abbasi, Gotam Das, Afsheen Maqsood, Abdul Razzaq Ahmed, Anand Marya, Mohammad Khursheed Alam, Artak Heboyan

**Affiliations:** 1 Department of Prosthodontics, Altamash Institute of Dental Medicine, Karachi 75500, Pakistan; 2 Prosthodontics Unit, School of Dental Sciences, Health Campus, University Sains Malaysia, Kota Bharu 16150, Malaysia; 3 Department of Prosthodontics, College of Dentistry, King Khalid University, Abha 61421, Saudi Arabia; 4 Department of Oral Pathology, Bahria University Dental College, Karachi 74400, Pakistan; 5 Department of Orthodontics, Faculty of Dentistry, University of Puthisastra, Phnom Penh 12211, Cambodia; 6 Department of Preventive Dentistry, College of Dentistry, Jouf University, Sakaka 72345, Saudi Arabia; 7 Center for Transdisciplinary Research (CFTR), Saveetha Institute of Medical and Technical Sciences, Saveetha Dental College, Saveetha University, Chennai 602105, India; 8 Department of Public Health, Faculty of Allied Health Sciences, Daffodil International University, Dhaka 1341, Bangladesh; 9 Department of Prosthodontics, Faculty of Stomatology, Yerevan State Medical University after Mkhitar Heratsi, Str. Koryun 2, Yerevan 0025, Armenia

**Keywords:** artificial intelligence, automated orthodontic diagnosis, deep learning, cephalometry, convolutional neural networks, head and neck imaging

## Abstract

This study aimed to analyze the existing literature on how artificial intelligence is being used to support the identification of cephalometric landmarks. The systematic analysis of literature was carried out by performing an extensive search in PubMed/MEDLINE, Google Scholar, Cochrane, Scopus, and Science Direct databases. Articles published in the last ten years were selected after applying the inclusion and exclusion criteria. A total of 17 full-text articles were systematically appraised. The Cochrane Handbook for Systematic Reviews of Interventions (CHSRI) and Newcastle-Ottawa quality assessment scale (NOS) were adopted for quality analysis of the included studies. The artificial intelligence systems were mainly based on deep learning-based convolutional neural networks (CNNs) in the included studies. The majority of the studies proposed that AI-based automatic cephalometric analyses provide clinically acceptable diagnostic performance. They have worked remarkably well, with accuracy and precision similar to the trained orthodontist. Moreover, they can simplify cephalometric analysis and provide a quick outcome in practice. Therefore, they are of great benefit to orthodontists, as with these systems they can perform tasks more efficiently.

## 1. Introduction

Artificial Intelligence (AI) is a term used to refer to neural networks of computers that imitate human intelligence. The AI’s key concepts are machine learning, representational learning, and deep learning (DL). Machine learning (ML) models include genetic algorithms, artificial neural networks, and fuzzy logic. These models can analyze data to perform various functions [1]. Representational learning and deep learning are subsets of ML in which the former requires a computer algorithm that analyzes the features required for classifying any data. The latter subset consists of artificial neural networks that mimic the human brain’s neural network, which is capable of deciphering features in a given model, such as a radiograph or an ultrasound [2]. The most demanding class of DL algorithms is the artificial neural network (ANN), which has the convolutional neural network (CNN) as its most popular subclass [3]. 

AI is becoming more prevalent in medicine and has reduced the need for humans to perform many tasks. Its applications in dentistry have also significantly evolved over the years [3]. AI algorithms support therapeutic decisions by assisting dentists in analyzing medical imaging and treatment planning. For example, it can be useful in identifying teeth and anatomical structures, detecting carious lesions, periapical lesions, and root fractures, and predicting the viability of dental pulp and the success of retreatment procedures [4]. Moreover, it has proven to be vital in the process of diagnosing head and neck cancer lesions, which is crucial in dental practice since early detection can greatly improve prognosis [5]. Briefly, it can be used to perform simple tasks in dental clinics without the involvement of a large numbers of dentists, resulting in accurate and comparable results. In addition, it is also widely used in dental laboratories and plays a significant role in dental education [3,4,5].

AI is advancing in the field of orthodontics. It is increasingly being used to interpret cephalometric radiographs and identify landmarks which help with the diagnosis and treatment planning of dentoskeletal discrepancies [6]. The most common types of AI architecture in orthodontics are ANN, CNN, and regression algorithms [6,7]. In addition, 3D scans and virtual models are beneficial in analyzing craniofacial or dental abnormalities. Aligners can be printed with 3D scan to formulate a data algorithm, which helps in providing and standardizing a specific treatment plan for the patients [4,7]. In machine learning-based studies, datasets are split into training and test sub-datasets, where the former is used to train the model and the latter is used to evaluate its performance on unseen data. In dentistry, there are different types of datasets; patient history, restorative and periodontal charts, results of diagnostic tests, radiographs, and oral images. These datasets can be inputted into models to generate outputs such as image interpretation, diagnosis, and future disease predictions [5,7].

The cephalometric landmarks are readily recognizable points representing hard or soft tissue anatomical structures. The structures are used as reference points for the identification of various cephalometric angles and cephalometric measurements [7]. The various cephalometric landmarks S (Sella), Po (porion), Pog (pogonion), Gn (gnathion), Go (Gonion), N (nasion), and Me (menton) are the most common hard tissue points. Whereas, A (most posterior tegmental point of the curvature of the maxillary sulcus) and B (the most anterior measure point of the mandibular apical base), P (pronasale), G (glabella) Sn (subnasale), Col (columella), LLA (lower lip anterior), and ULA (upper lip anterior) are common soft tissue points used in cephalometric analysis [8]. Several methods have been used to automatically identify these landmarks for a very long time. The approaches that have been tried and tested include pixel intensity, knowledge-based methods, and template matching [5,6]. However, the results were not always satisfactory. In recent years, deep learning algorithms (AI) have been widely introduced to detect landmarks automatically and accurately on lateral cephalograms [7]. Recent studies on automatic cephalometric landmark identification using deep learning methods demonstrated improved detection accuracy when compared with other machine learning methods [7,8,9]. Monill-González et al. conducted a study to compare the performance of one of the deep learning methods, You-Only-Look-Once (YOLO v3), with human examiners and found promising results. YOLO is known to take a shorter amount of time to identify landmarks. Ji-Hoon Park compared YOLOv3 and Single Shot Multi-Box Detector (SSD) and found YOLOv3 to be the more promising method for identifying automated cephalometric landmarks [8]. Despite this, only few studies on the AI performance of cephalometric analysis have proven beneficial to dentists. A large number of skeletal and soft tissue landmarks are required to evaluate and predict the outcome of a disease [9]. For a better understanding of the application of these methods in clinical orthodontics, more results of cephalometric analysis need to be obtained. While landmark identification is an essential part of the diagnostic process, image-related errors and expert bias can influence the results. It is therefore required to design a study to assess whether AI can achieve similar results to clinicians in cephalometric landmark detection upon repeated detection trials. One might expect improved performance with a substantial amount of learning data, but manually detecting multiple landmarks would be challenging [10]. 

This study aimed to provide an overview of the existing literature on how far artificial intelligence is being used to support the identification of cephalometric landmarks. It is hypothesized in this study that AI accurately identifies cephalometric landmarks compared with human examiners and other machine learning methods.

## 2. Materials and Methods

### 2.1. Focused Question

This systematic review was conducted using PRISMA-DTA (Preferred Reported Items for Systematic Review and Meta-Analysis-Diagnostic Test Accuracy) guidelines [11]. Our intended question was “Does AI play a significant role in measuring cephalometric landmarks accurately as compared to the human examiner?” The question was constructed according to the Participants Intervention Comparison Outcome and Study (PICOS) strategy [12].

#### 2.1.1. Population

Patients’ lateral cephalometric radiographs, three-dimensional (3D), were taken using OrthoCeph^®^ OC100 (GE Healthcare, Finland), Carestream CS 9000 3D unit, PA-cephalograms were synthesized from cone-beam-computed tomography scans (CBCT-PA) taken with a Planmeca (Prolin XC) X-ray unit, and digital images (computed radiography) of the cephalometric radiographs were taken using CEFBOT (an artificial intelligence (AI)-based cephalometric software) (RadioMemory Ltd., Belo Horizonte, Brazil).

#### 2.1.2. Intervention

AI techniques (machine learning; deep learning, CNN, ANN, PANN) were applied in orthodontics concerning the cephalometric analysis, and the modifications were made with commercial cephalometric analysis software (V-Ceph version 8).

#### 2.1.3. Comparison

The comparison was made based on automatic algorithm architects, testing models, lateral cephalometric radiograph analysis, rater opinions, machine-to-orthodontist comparison, success detection rate (SDR), Single Shot Multibox Detector (SSD), and Landmark error (LE) value calculation.

#### 2.1.4. Outcome

For the association between AI and human findings, bland Altman plots were used to measure outcomes such as sensitivity, specificity, and intraclass correlation coefficient (ICC). 

#### 2.1.5. Study Design Type

For this review, we considered clinical trial-based studies published in English.

### 2.2. Eligibility Criteria

Two examiners evaluated the articles (N.J and N.K) according to the following inclusion criteria:

1. Articles with AI-based cephalometric analysis for landmark identification,
2. Clinical trials,
3. English language articles.

Review articles, letters to editors, gray literature, case reports, incomplete articles which showed only the abstract without a definitive comparison between AI and human examiners, articles in which there was no comparison of AI with human examiners, AI not related to orthodontics, AI in orthodontics not related to cephalometry and non-deep-learning methods (e.g., knowledge- or atlas-based articles or those involving shallow machine learning) were excluded.

### 2.3. Search Methodology

An electronic search was carried out with PubMed/MEDLINE, Google Scholar, Cochrane, Scopus, Science Direct, and research databases. The medical subject heading (MeSH) and other keywords used in the articles were “intelligence, machine”, “machine intelligence”, “artificial intelligence”, “computational intelligence”, “classification”, “orthodontics”, “cephalometry”, “learning, deep”, “algorithms”, “neural networks, computer”, and “expert systems”. The articles published in the last decade (2010 to 2021) were included. The last search was performed in October 2021. Two well-calibrated reviewers (N.J. and N.K.) performed the search. Consensus resolved disagreements, and a third examiner (N.A.) was consulted. All the titles and abstracts were read thoroughly from the articles searched primarily, and irrelevant studies were excluded. The relevant articles were listed and scrutinized for any similar studies that matched our inclusion criteria. We read the full texts of the included studies to obtain appropriate results, and the findings were recorded.

### 2.4. Quality Assessment of Included Studies

The quality assessment was conducted according to the parameters described in the Cochrane Handbook for Systematic Reviews of Interventions [13]. The quality of each study was classified into low, medium, and high risk of biasness. The same 2 review authors autonomously sort out the search to amplify the number of studies recovered. The reviewers surveyed each selected article according to the inclusion criteria and directed unbiased evaluation, and any ambiguity was settled by consultation with a third reviewer (N.A.).

Furthermore, the Newcastle–Ottawa quality assessment scale (NOS) was used for the analysis of the included articles [14]. The analysis was based on the three core quality analysis parameters: case and group (definition, selection, and representativeness), comparability (comparison of case and control groups; analysis and control of confounding variable), and exposure (use of a universal assessment method for both control and case groups; dropout rate of patients in the included studies). A star system was implemented for rating the included studies. The quality of each study was classified into low, moderate, or high risk of biasness.

## 3. Results

### 3.1. Search Results

**Figure 1:**
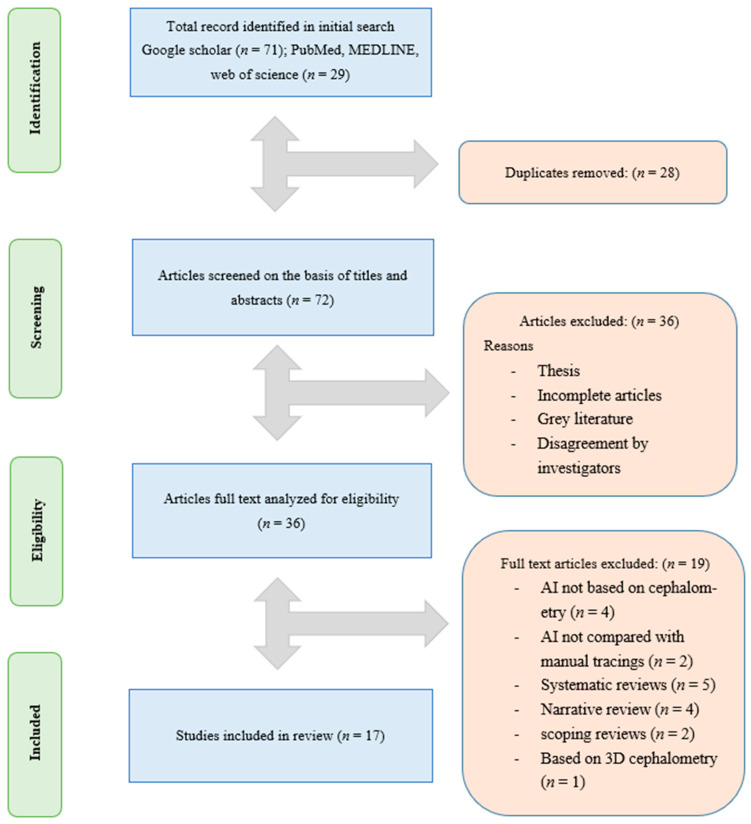
The PRISMA flow diagram for the literature search performed in this study.

The title search yielded 100 articles from 2010 to 2021, from which we removed 28 duplicates, and 36 entries that did not analyze AI. Thirty-six articles were selected for full-text reading, which lead to a further exclusion of 19 articles based on the inclusion criteria. A total of 17 full-text articles were included in this systematic review, as shown in Figure 1.

### 3.2. General Analysis and AI with Human Comparison in Included Studies

All 17 included studies [15,16,17,18,19,20,21,22,23,24,25,26,27,28,29,30,31] were clinical trial-based studies. The deep learning-based AI technology was used for assessment in all 17 studies included, with YOLO version 3 (*n* = 2) and CNN (*n* = 12), Res Net 50 (*n* = 1), and FALA (*n* = 1), and PANN (*n* = 1) being the most common algorithms adopted. Moreover, human examiners were included for comparative analysis in all included studies. The lateral cephalography was used to identify landmarks by human examiners and AI machines in all included studies. The control group for the reference test was recognized by 2 orthodontists in 10 studies, 12 orthodontists in 1 study, 1 orthodontic expert in 3 studies, and 3 orthodontists in 1 study. Additionally, 14 studies [15,16,17,18,21,22,23,24,25,26,27,29,30,31] proposed that AI-based automatic cephalometric analyses provide clinically acceptable diagnostic performance, whereas 3 studies [19,20,28] reported that there is no difference between human gold standard techniques and the AI’s predictions. Overall, the AI algorithm architecture (CNN, ANN) showed promising results in cephalometric landmark detection and analysis compared with human subjects, as shown in Table 1. Moreover, 19 articles [5,6,8,9,10,32,33,34,35,36,37,38,39,40,41,42,43,44,45] were excluded from this review, as shown in Supplementary Table S1.

### 3.3. Quality Assessment Outcomes

According to CHSRI, 14 studies mentioned choosing their patients randomly and 2 mentioned blinding their participants or assessors. Eight studies mentioned the withdrawal/dropout of their participants. All 17 studies repeated the measurement of their variables. Likewise, two studies carried out sample size estimation. All included studies reported their outcomes and examiner reliability. 

Furthermore, twelve studies were categorized as having a “moderate” risk of bias and five studies were categorized as “low” risk of bias (Table 2).

In accordance with NOS, the included studies scored in the range of 5 to 9 points, with a mean score of 6.52. Fourteen studies reported a “moderate” risk of bias, while two studies a high risk of bias and one study showed low risk of bias (Table 3).

## 4. Discussion

AI technologies are radically transforming various aspects of dentistry. The use of AI in orthodontics has also grown significantly in the last decade with improvement in diagnostic accuracy, treatment planning, and prognosis prediction. This systematic review was carried out to evaluate the performance of AI for the detection of cephalometric landmarks with an accuracy and precision similar to an orthodontist [1,2].

Cephalometric analysis is carried out to identify various landmarks or points on the radiograph that helps in establishing various relationships and planes, which in turn aids in establishing the diagnosis and treatment plan. Manual analysis is time-consuming and accompanied by a possibility of significant inter-observer variability. Over the past 40 years, researchers have introduced and suggested various methods of AI for cephalometric landmark identification. Initially, they did not seem to be accurate enough for use. However, with time, newer algorithms were introduced with which the increased computational power provided enhanced accuracy, reliability, and efficiency [3,4,10]. 

Previously, knowledge-based techniques or image-based learning systems were used to automate landmark identification. However, recent studies have focused on deep learning AI systems. In this systematic review, the majority of the included studies created an automated cephalometric analysis using a specialized CNN-based algorithm [15,17,18,19,20,21,22,23,24,25,27,28,31]. Among these, few studies demonstrated conflicting results as certain landmarks and analyses were not accurately identified by the AI system, i.e., saddle angle, Mx In–NA line, Mn In-NB line [15], lower incisor root tip [21], SN-MeGo [19], porion, orbitale, PNS, and gonion [21]. It could be because certain landmarks, such as porion, orbitale, and PNS, are hard to detect due to surrounding superimposing anatomical structures. Moreover, as some other landmarks exist bilaterally, they might cause errors in the process of determining the midpoint of those bilateral structures [18]. Kim MJ et al. [22] further added that AI prediction is affected by the expert examiner’s identification pattern. If the examiner shows difficulties in some areas, the AI predictions will reflect these difficulties as the CNN model emulates the human examiner’s landmark identification pattern and performs prediction. 

Moon et al., [18], Hwang et al., [20], and Hwang et al., [21] compared YOLO v3, a real-time object detection algorithm, with human readings, and found that AI can identify cephalometric landmarks as accurately the human examiners. Mario et al. [16] also found equivalent results as compared to human experts, although their work was based on PANN. Similarly, Kim YH et al. [17] found that the deep learning method achieved better results than the examiners for the detection of some cephalometric landmarks, especially those that are located anatomically on curves. These landmarks are sensitive to identification errors because of reduced human-induced variability, which, in turn, is a result of certain factors including the overall knowledge of the examiners about the subject and the quality of cephalometric images. Moreover, according to Moon et al. [18] and Kunz et al. [19], an adequate amount and quality of data are needed to create an accurate and clinically applicable AI. Moon et al. [18] further reported that if we take the inter-examiner difference of 1.50 mm between human examiners into consideration, the estimated quantity of learning data seemed to be at least 2300 data sets. Similar thoughts were shared by Song et al. as well [24]. He added that human beings’ skeletal anatomies are so different that if sufficient data is not included in the training dataset, the results might be rambling. Strikingly, the minimum amount of learning data calculated by Moon et al. [18] far outnumbered the learning data (40–1000) that were included in previous studies, thus reporting conflicting results [22,23,24,25,27,28,29,30,31].

Moreover, the quality of the data also plays an important role. Lee et al. [23] used a public database and it was observed that even though the overall landmarks were located within acceptable margins from ground truth, the detected landmarks and ground truth did not adequately match, which could be owing to the following reasons: (1) the input images were scaled from 1935 × 2400 pixels to 64 × 64 pixels so that the fine error in the scaled images grew rapidly as the images were enlarged to the original size, and (2) the regression systems were trained without proper use of deep learning-related techniques. Furthermore, certain studies used the datasets presented in the “International Symposium on Biomedical Imaging (ISBI) 2014 and 2015 grand challenges in dental x-ray image” [23,24,27,30,31]. This dataset was somewhat flawed as it used a smaller sample size with a wide age range (six to 60 years). Moreover, the mean intra-observer variability of the two experts was 1.73 and 0.90 mm, respectively, which is very high. Thus, there were chances of unnecessary bias in the trained model, which suggests uncertainty with the clinical applicability of this dataset.

Unlike others, Kunz et al. [19] used high-quality cephalometric radiographs that had been generated on an approved X-ray unit and not collected from the public domain. The radiographs were not selected beforehand so that a vast variety of different skeletal and dental problems were included, which is a prerequisite for reliable AI learning. In addition, only experienced practitioners were asked to perform landmark identification and tracing, which resulted in a very high intra-rater and inter-rater reliability. Kunz et al. [19] also showed that the measurements are in good agreement concerning the Bland–Altman plots.

Lastly, the literature shows that the recent deep learning-based techniques have outperformed the conventional machine learning-based techniques in terms of accurate tracing of cephalometric landmarks. Kim H et al. [26] achieved a maximum accuracy of 96.79%. In addition, Kim YH et al. [17] found that the deep learning method achieved better results than the examiners for the detection of some cephalometric landmarks. With such promising results it would not be wrong to say that, with the continuous development and advancements, AI could shortly exceed manual markings performed by clinical experts, consequently saving labor, time, and effort.

The review had a few shortcomings; some of the included studies suffered from a range of risks of bias and few studies utilized similar datasets. There were several studies in which there was no clarification on how the human annotator labels led to test datasets. Certain studies employed the use of only a single expert, which could have affected the results because of variations in landmark identification. Additionally, there were very few studies that employed the use of independent datasets. Studies conducted in the future should consider using wider outcome sets and aim at testing deep-learning applications across different settings.

## 5. Conclusions

The results from the various articles analyzed in this systematic review suggest that the applications of artificial intelligence systems are promising and reliable in cephalometric analysis. Despite the limitations, almost all of the studies agreed that AI-based automated algorithms have worked remarkably well, with accuracy and precision similar to trained orthodontists. It can simplify cephalometric analysis and provide a quick outcome in practice, which can save practitioners time and enhance their performance. Additionally, it can be of greater benefit and used as an ancillary support for orthodontists and clinicians with lesser experience.

## Figures and Tables

**Table 1:** Characteristics of the selected studies (*n* = 17).

Author and Year	Study Design	Groups		Assessment Method	Studies Accuracy	Outcome	Recommendations and Comments
		Study	Control				
Sangmin Jeon 2021 [15]	Retrospective study/clinical trial	Automatic cephalometric analysis using CNN	Orthodontist with over 7 years of experience	The measurements from 35 patients obtained through a conventional and Ceph-X (AI program) were analyzed with the help of paired t-tests and Bland–Altman plots	There was a difference in dental and skeletal measurements	CNN-based AI diagnostic analysis was clinically acceptable	It was recommended that the dental measurement needs manual adjustments for precision and adequate performance
Mario et al., 2010 [16]	Clinical Trial	Paraconsistent artificial neural network (PANN)	Three orthodontists	The cephalometric analysis was carried out with AI-based system (PANN) and three expert orthodontists	The accuracy was carried out with the kappa index, which revealed a moderate to perfect agreement	The AI-based analysis provided equal outcomes to expert orthodontists	PANN was endorsed as a promising tool in cephalometric analysis
Kim et al., 2021 [17]	Clinical Trial	Automatic cephalometric analysis using CNN	Two trained orthodontists	Three skeletal landmark analyses were performed on 950 cephalographs through CNN and trained orthodontists’ opinions	CNN-based SDR values ranged from 47.3% to 66.7%, whereas the SDR value for orthodontists was in the range of 3.35% to 7.3%	The CNN deep learning showed promising results compared with human examiners	In future studies, the examiner variability analysis should be considered for clinical applicability
Moon et al., 2020 [18]	Clinical Trial	Deep-learning method, a modified you-only-look-once version 3 algorithm	1 Orthodontist examiner	2200 images were first used to train AI machines on about 80 anatomical landmarks, out of 2400 cephalography images. The remaining 200 images were used for comparisons with human examiners	NM	The accuracy of AI was accurate compared with human opinions	It was proposed that at least 2300 images are needed to train AI machines accurately for future applications
Kunz et al., 2019 [19]	Clinical trial	CNN deep learning algorithm	12 experienced orthodontists	The AI machine was trained with 1792 images of 18 cephalometric landmarks. Later, 12 orthodontic landmarks on 50 images were analyzed	The Pearson correlation (*r* > 0.864; *p* < 0.001) was found between the AI system and orthodontist opinion	The accuracy of AI and orthodontist opinion was found similar	The presented analysis can be expanded either by new geometrical calculations using the already existing landmarks or by retraining the AI algorithm with new ones
Hye et al., 2021 [20]	Clinical trial	AI-based on the YOLO version 3 algorithm	2 certified orthodontists	The AI detected 19 landmarks were compared with orthodontists’ identified landmarks	The value of SDR in AI with 2 mm of error was observed to be 75.5% and the SCR value was 81.5% it was similar to the values obtained from orthodontists	The orthodontists and AI showed similar findings	It is proposed that AI can sustain and improve its efficiency under the supervision of orthodontists
Hye et al., 2019 [21]	Clinical trial	AI-based on YOLOv3	2 orthodontists	The comparison was performed on 80 anatomical landmarks in 283 images between orthodontists and AI machine	The detection of landmarks identification error was less with AI in a range of 0.9 to 1.2 mm	The accuracy of landmark identification was slightly more with the AI system compared to orthodontists	AI systems should be used as a supplementary tool along with orthodontist expertise in the diagnostic process to improve reliability
Kim et al., 2021 [22]	Clinical trial	CNN	1 Orthodontist	The cephalometric landmarks findings of orthodontists from 85 CBCT images were compared with AI reading	The AI system accuracy was MRE 2.23 ± 2.02 and SDR = 60.88%, compared to human recording	The CNN model showed better consistency than orthodontist experts after repetitive landmark identification rounds	The repetitive manner of application AI might work superior to human examiners
Hansang et al., 2017 [23]		A CNN-based landmark detection system	2 orthodontists	The data on 19 landmarks were first manually marked by an orthodontist, for AI 150 images were used for training and testing	The overall landmark accuracy was considered within margins from the human findings without heavy outliers	The CNN showed adequate performance and successfully locates the landmarks with substantial margins	Future works can include further improvement by using deeper network structures and extension of our framework to other landmark detection problems
Song et al., 2021 [24]	Clinical trial	A CNN-based landmark detection system	2 orthodontists	The ROI patches were first cropped from training images, and then the CNN-based ResNet 50 system was used to detect landmarks from the patches.	Performance with CNN was good in landmarks While the accuracy was low in other landmarks	The SDR and SCR values of CNN were better than the benchmark identified	A global context information should be utilized in the future to identify the performance of AI
Arık et al., 2017 [25]	Clinical trial	Deep learning CNN	2 orthodontic experts	The CNN architect was used for the identification of landmarks on 250 images after training the AI system on 150 images. The findings were then compared with manual orthodontist recordings	The overall SDR accuracy within a range of 2 mm error was 67.68 to 75.58%	The study revealed a high success detection rate compared with the benchmark values in the literature	Future outcomes could be improved by increasing the size and diversity of AI machine training sessions
Kim et al., 2020 [26]	Clinical trial	Deep learning web-based application	2 expert orthodontists	The 2075 cephalographs from two institutes were analyzed for 23 landmarks. The AI machine was first trained with a stacked hourglass deep learning model for landmark detection in images.	The SDR of the deep learning model was 1.37 ± 1.79 mm	The automated cephalometric landmark detection with a web-based application approach is promising. It saves time and effort compared to manual marking	NM
Lee et al., 2020 [27]	Clinical trial	CNN	2 orthodontic experts	The AI algorithm analysis was based on a region of interest (ROI) patch extracted from cephalometric images obtained from ISBI 2015 grand challenge	The CNN architect showed: Mean (LE) = 1.53 ± 1.74 mm and SDR value of 82.11 to 95.95%	The CNN architect provides adequate landmark detection outcomes and can be utilized clinically as a computer-aided detection tool and a means of education	In the future, various medical centers, races, and regions are recommended for use to develop an AI model that has a wider application
Nishimoto et al., 2019 [28]	Clinical trial	CNN	1 orthodontist	The AI-based CNN model was trained first on 153 images, and then testing of ten skeletal landmarks was performed on 66 images; later on, the outcome was compared with orthodontist recordings	The landmark error was 16.22 to 17.02 pixels. The average SSR was similar to manual readings	The CNN model utilizing web-based cephalogram images is an ideal approach for skeletal landmark detection	The approach adopted in this study is recommended for the detection of other skeletal landmarks in the face in dentistry
Lindner et al., 2016 [29]	Clinical trial	Random forest, machine learning	2 orthodontists	The AI-based FALA system was used to identify 19 skeletal landmarks on 400 cephalograms before comparison with orthodontists’ finding	The skeletal landmarks were identified with an SDR value of 84.7% with a landmark error of 1.2 mm pixels	The clinically acceptable precision range was 2 mm. The FALA model identifies skeletal cephalometric landmarks accurately and rapidly; this system can improve the diagnosis of orthodontic patients	NM
Wang et al., 2018 [30]	Clinical trial	Machine learning; CNN, Decision tree regression	2 orthodontists	The AI machine was used to detect landmarks on 300 images from the 2015 ISBI challenge and separate 165 images from the clinical database of the institute	AI detects landmarks up to an SDR value of 72% with a landmark error of 2 mm	The CNN-based machine learning performed exceptionally well in landmark detection	The method complexity was greater, which needs to be further evaluated to ease the procedure for the masses
Oh K et al., 2020 [31]	Clinical trial	CNN	2 orthodontists	AI system was used to detect landmarks on 400 images. The AI model was first trained with 150 images, then the outcome was validated on further 150 images, and, finally, the test was carried out on 100 radiographs before comparison with the orthodontist readings	The EDR value of the CNN model was in the range of 13.80% to 24.11% with a LE value of 2 mm in various tested datasets	The CNN model was accurate in landmark detection in normal and distorted cephalogram images	NM

NA: Not applicable, NM: Not mentioned, AI: Artificial Intelligence, ICC: intraclass correlation coefficient, CNN: Convolutional neural networks, ANN: Artificial neural networks, PANN: Paraconsistent artificial neural network, 3D: three Dimensional, 2D: Two-dimensional, DL: Deep learning, CAD/CAM: Computer added design computer added manufacturing, PA: Periapical radiograph, CBCT: Cone-beam computerized tomography, ML: Multiple landmarks, SDR: success detection rate, SSD: Single Shot Multibox Detector, YOLOv3: You-Only-Look-Once version 3, T1: image taken at the first visit, T2: after the observation period, FALA: fully automatic landmark annotation, NM: not mentioned, LE: landmark error, EDR: Endpoint detection and response.

**Table 2:** Methodological quality assessment results of the included studies (*n* = 17).

Author and Year	Randomization	Blinding	Withdrawal/Dropout Mentioned	Variables Measured Many Times	Sample Size Estimation	Selection Criteria Clear	Examiner Reliability Tested	Expected Outcomes Prespecified	Quality of Study/Bias Risk
**Sangmin Jeon** **2021** [15]	Y	UC	N	Y	N	Y	Y	Y	Moderate
**Mario et al., 2010** [16]	Y	UC	Y	Y	N	UC	Y	Y	Moderate
**Kim et al., 2021** [17]	Y	Y	Y	Y	Y	UC	Y	Y	Low
**Moon et al., 2020** [18]	Y	UC	Y	Y	UC	Y	Y	Y	Low
**Kunz et al., 2019** [19]	Y	UC	Y	Y	UC	Y	Y	Y	Low
**Hye et al., 2021** [20]	UC	UC	Y	Y	N	Y	Y	Y	Moderate
**Hye et al., 2019** [21]	UC	UC	N	Y	N	Y	Y	Y	Moderate
**Kim et al., 2021** [22]	Y	UC	Y	Y	Y	Y	Y	Y	Low
**Hansang et al., 2017** [23]	Y	N	N	Y	UC	Y	Y	Y	Moderate
**Song et al., 2021** [24]	Y	N	Y	Y	N	N	Y	Y	Moderate
**Arık et al., 2017** [25]	Y	N	N	Y	N	UC	Y	Y	Moderate
**Kim et al., 2020** [26]	Y	N	N	Y	N	Y	Y	Y	Moderate
**Lee et al., 2020** [27]	Y	N	Y	Y	N	Y	Y	Y	Low
**Nishimoto et al., 2019** [28]	Y	Y	N	Y	N	UC	Y	Y	Moderate
**Lindner et al., 2016** [29]	Y	N	UC	Y	N	Y	Y	Y	Moderate
**Wang et al., 2018** [30]	Y	N	UC	Y	N	N	Y	Y	Moderate
**Oh K et al., 2020** [31]	N	N	UC	Y	UC	N	Y	Y	Moderate

A study was graded to have a low risk of bias if it yielded six or more “yes” answers to the nine questions, moderate risk if it yielded three to five “yes” answers, and high risk if it yielded two “yes” answers or less; Y: yes, N: no, UC: unclear.

**Table 3:** Newcastle–Ottawa scale-based quality assessment of the selected studies (*n* = 17).

Author and Year	Selection	Comparability	Exposure	Newcastle– Ottawa Quality (Total)
**Sangmin Jeon 2021** [15]	****	**	***	Low
**Mario et al., 2010** [16]	****	*	***	Moderate
**Kim et al., 2021** [17]	****	*	**	Moderate
**Moon et al., 2020** [18]	***	*	**	Moderate
**Kunz et al., 2019** [19]	***	*	***	Moderate
**Hye et al., 2021** [20]	***	*	**	Moderate
**Hye et al., 2019** [21]	**	*	**	High
**Kim et al., 2021** [22]	****	*	***	Moderate
**Hansang et al., 2017** [23]	****	*	**	Moderate
**Song et al., 2021** [24]	***	**	**	Moderate
**Arık et al., 2017** [25]	****	*	**	Moderate
**Kim et al., 2020** [26]	***	**	**	Moderate
**Lee et al., 2020** [27]	***	*	***	Moderate
**Nishimoto et al., 2019** [28]	**	**	***	Moderate
**Lindner et al., 2016** [29]	***	**	**	Moderate
**Wang et al., 2018** [30]	**	**	***	Moderate
**Oh K et al., 2020** [31]	**	**	*	High

A total of nine stars can be awarded to a study. Any study with the maximum stars was rated as having a low risk of bias. A study with six to eight stars was declared as having moderate bias, whereas a study with five stars or less was considered as having a high risk of bias.

## Data Availability

Data related to the study can be provided by the authors on reasonable request.

## Supplementary Materials

The following supporting information can be downloaded at: https://www.mdpi.com/article/10.3390/healthcare10122454/s1, Table S1 Methodological list of studies excluded from this review and reasons for exclusion (*n*= 19).
