# Supplementary material for: Development, Application, and Performance of Artificial Intelligence in Cephalometric Landmark Identification and Diagnosis: A Systematic Review
Source: Healthcare (Basel). 2022 Dec 5;10(12):2454. doi: 10.3390/healthcare10122454 (PMC9778374; doi:10.3390/healthcare10122454)
Supplement: Supplementary file 1 [file healthcare-10-02454-s001.zip › healthcare-2024687-supplementary.pdf]

healthcare

MDPI

Table S1. Methodological list of studies excluded from this review and reasons for exclusion (*n*= 19).

Author year	Reason of exclusion
Wang et al,2015[5]	A narrative review
Shahidi S et al, 2013[6]	AI-based but not related to cephalometry
Park et al 2019[8]	AI tracings on cephalogram not compared with manual tracings
Monill et al 2021[9]	A scoping review
Mamta et al 2021[10]	A narrative review
Schwendicke et al 2021[32]	A systematic review
Sanjeev et al 2021[33]	A systematic review
Sanjeev et al 2021[34]	A systematic review
Kuofeng et al 2019[35]	A systematic review
Bichu et al 2021[36]	A scoping review
Silva et al 2021[37]	A short communication
H. Alqahtani 2019[38]	Not compared with the human experts
Leonardi et al, 2008[39]	A narrative review
Mihee et al 2021[40]	AI-based but not related to cephalometry
Mani et al 2020[41]	A systematic review
Akdeniz et al 2021[42]	A narrative review
Shin et al 2021[43]	AI-based but not related to cephalometry
Faure et al 2016[44]	Based on 3D cephalometry
Yu et al 2020[45]	AI-based but not related to cephalometry
